# Best Case/Worst Case Communication Tool for Trauma Intensive Care Units

**DOI:** 10.1001/jamasurg.2025.3782

**Published:** 2025-09-24

**Authors:** Melanie L. Fritz, Alexandra H. Hernandez, Amy B. Zelenski, Jenna Nitkowski, Carly Sobol, Kristine Kwekkeboom, Taylor Bradley, Jolene Tsang, Kyle Bushaw, Alex Dudek, Lily Stalter, Margaret L. Schwarze

**Affiliations:** 1 Department of Surgery, University of Wisconsin–Madison, Madison; 2 Department of Surgery, University of Washington, Seattle; 3 Department of Medicine, University of Wisconsin–Madison, Madison; 4 Center for AIDS Intervention Research, Medical College of Wisconsin, Milwaukee; 5 School of Nursing, University of Wisconsin–Madison, Madison; 6 School of Medicine and Public Health, University of Wisconsin–Madison, Madison; 7 Public Health Madison and Dane County, Madison, Wisconsin

## Abstract

**Importance:**

Advanced communication techniques can support seriously injured older adults facing a significant change in health trajectory or functional status. Optimal use of these techniques requires effective implementation among trauma team members in intensive care units (ICUs).

**Objective:**

To evaluate implementation of the Best Case/Worst Case-ICU (BC/WC-ICU) communication tool.

**Design, Setting, and Participants:**

This quality improvement study involved implementing the BC/WC-ICU in the context of a randomized clinical trial. Each site received 3 months of implementation training during 1 of 4 sequential waves from October 2023 to September 2024; ongoing implementation was evaluated until January 2025. Participants included such trauma team members as attendings, fellows, residents, advanced practice providers, and bedside nurses at 8 high-volume trauma centers across the United States.

**Intervention:**

BC/WC-ICU is a communication tool used daily on rounds that includes team discussion of major 24-hour events and of the best- and worst-case scenarios for recovery, which are annotated on a graphic aid. Clinicians use the graphic aid to discuss prognosis with patients and families.

**Main Outcomes and Measures:**

Reach, Effectiveness, Adoption, Implementation, and Maintenance (RE-AIM implementation outcomes) of the intervention.

**Results:**

Two hundred eight trauma surgeons, intensivists, and fellows completed 1-on-1 training, and the intervention reached an estimated 1300 patient families. Clinicians reported the intervention effectively supported families through consistent messaging about prognosis that improved downstream decision-making and reduced moral distress. Mean (SD) site adherence ranged from 45% (30.4) to 100% (0), and graphic aid fidelity was high, with sites scoring a mean (SD) 6.22 (2.02) to 7.12 (1.39) on an 8-point rubric. Implementation was hindered by competing clinical tasks, fear of communicating prognosis, misunderstanding the tool, and a belief that BC/WC-ICU was not innovative, which generated hesitance about the intervention’s utility. Long-term use of the intervention lagged at 12 months with the exception of 1 trauma center.

**Conclusions and Relevance:**

This study found that implementation of BC/WC-ICU in trauma ICUs is feasible, supports prognostic communication, and can improve the clinician-family relationship. Future efforts to advance clinician-patient communication will need to consider identified barriers, including the rapid pace and high acuity of critical care and disincentives to prioritize communication.

## Key Points

**Question:**

What are the outcomes of implementing an intervention to improve communication for seriously injured older adults?

**Findings:**

This quality improvement study involved implementing the tool Best Case/Worst Case-ICU with adherence and fidelity at 8 trauma centers, reaching more than 1300 seriously injured older adult patients. The tool effectively supported clinical teams and, by clinician report, families through consistent prognostic communication, although implementation efforts faced logistical and attitudinal barriers.

**Meaning:**

Best Case/Worst Case-ICU can be implemented in the trauma ICU to support prognostic communication; future efforts to advance clinician-patient communication need to consider identified barriers, including the high acuity of critical care and disincentives to prioritize communication.

## Teaser

This quality improvement study implements a tool supporting prognostic communication in 8 trauma intensive care units and assesses the reach, effectiveness, adoption, implementation, and maintenance outcomes among clinicians.

## Introduction

Millions of older adults sustain traumatic injuries every year in the United States.^1,2^ In addition to having higher rates of mortality, older adults who survive serious injuries often experience a significant alteration in health trajectory and a major change in functional status.^3^ Advanced communication techniques support patients who have suffered serious injuries by helping them understand the prognosis and promoting goal-concordant care.^4,5^ While innovations to improve communication have promise,^6,7,8,9^ their impact depends on clinicians to use such techniques as intended.^10,11^

We developed Best Case/Worst Case to deliver prognostic information to support patients and families as they anticipate and plan for possible futures. Initially developed for treatment decision-making, this tool has appeal across a variety of specialties,^12,13,14,15^ with broad interest in leveraging these techniques in the trauma intensive care unit (ICU).^16,17,18,19^ Because decisions about trauma surgery are typically emergent, we adapted the original tool for downstream use, illustrating how new events change the clinical trajectory. Daily updates support communication over time as information about physiologic response to injury and potential for functional recovery becomes apparent.^20,21^

The objective of this study is to evaluate the implementation of Best Case/Worst Case-ICU (BC/WC-ICU) at 8 major US trauma centers. We used normalization process theory to guide data collection and analyzed the Reach, Effectiveness, Adoption, Implementation, Maintenance (RE-AIM) outcomes to describe the achievements and challenges associated with efforts to improve serious illness communication.^22,23,24,25^

## Methods

### Study Design

We delivered BC/WC-ICU training as a quality improvement initiative^26^ within a stepped-wedge randomized clinical trial in accordance with Standards for Quality Improvement Reporting Excellence (SQUIRE) reporting guidelines.^27,28,29^ Each site received 3 months of implementation training during 1 of 4 sequential waves. Subsequently, sites were expected to use the intervention routinely with ongoing implementation support. The University of Wisconsin institutional review board approved this study. Study sites ceded review according to single-site institutional review board standards.

### Setting and Participants

Eight high-volume level I trauma centers accredited by the American College of Surgeons in New England, the Mid-Atlantic, Southeast, Midwest, and Pacific Northwest United States participated. We aimed to train all members of the trauma team, including attendings, fellows, residents, advanced practice providers (APPs), and bedside nurses. Clinicians were asked to use the intervention with all trauma patients 50 years and older with an anticipated ICU stay of at least 3 days; they could also use BC/WC-ICU for patients who did not meet these criteria.

### Intervention

**Figure 1.**
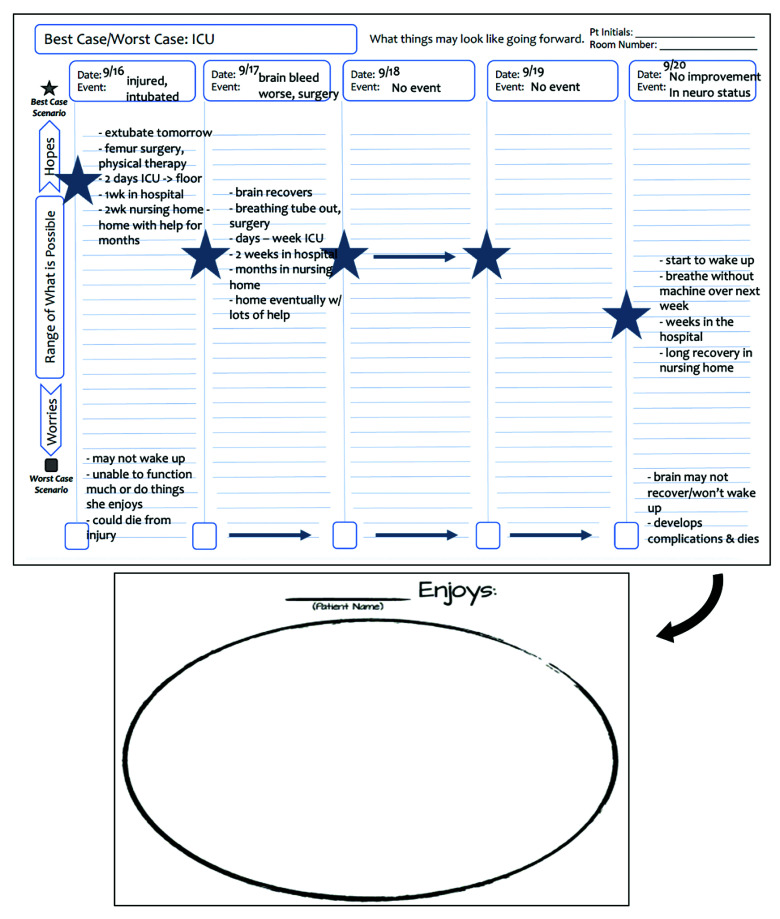
Sample Best Case/Worst Case-ICU Graphic Aid This graphic aid demonstrates 5 days in the trauma intensive care unit (ICU). A new event or lack of event is noted each day. When events change the best-case scenario, the best-case star changes position and a new story is noted (9/16, 9/17, 9/20). When there have been no significant events, the star representing the best-case scenario remains in the same location (9/18, 9/19), and the story is carried forward with an arrow. Written notes describing the worst-case story are updated on an as-needed basis. The reverse side of the graphic aid includes a prompt for families to share information about what the patient enjoys, to help remind the family and care team what is important to the patient.

The BC/WC-ICU intervention is used daily on rounds. The team leader notes any major, trajectory-altering events over the past 24 hours and describes the best- and worst-case narratives given the current treatment approach. Concurrently, another team member completes the graphic aid to establish a shared prognostic narrative (Figure 1). The graphic aid is hung in or near the patient’s room so any clinician can use the graphic aid with the language of “Here’s what we are hoping for” and “Here’s what we are worried about” to discuss the best- and worst-case scenarios with patients and families.^20,21^

### Implementation

The implementation team included an attending surgeon (M.L.S.), 3 surgical residents (M.L.F., A.H.H., C.S.), and a researcher with a doctorate in education (A.B.Z.). In advance, we met with the site principal investigator (PI), APPs, nursing leadership, and an on-site liaison (surgical resident or APP) who would provide implementation support. We discussed local processes of ICU care to understand contextual factors and drafted plans to address anticipated barriers.

Our team conducted an on-site visit to observe patterns of care, deliver targeted training to clinicians, and provide in-person coaching as ICU teams began using BC/WC-ICU. We maintained weekly communication with the site liaison to address implementation barriers. After 3 months, the team returned to assess performance and provide additional coaching.

After implementation, the liaison continued to support the ICU team by attending rounds weekly and troubleshooting barriers. The implementation team generated monthly feedback messaging (eFigures 1 and 2 in Supplement 1), which was shared via email to encourage high-quality use of BC/WC-ICU.

### Clinician Training

We tailored training to the clinical roles of clinicians who would interact with BC/WC-ICU (eTable 1 in Supplement 1). For attendings and fellows, we provided 30-minute 1-on-1 training targeting the skill of scenario planning to describe the interplay between a new clinically significant event and the best-case scenario,^30,31^ with summative assessment on training completion. For residents and APPs, we delivered group training to complete the graphic aid on rounds and use the graphic aid with the phrases “we are hoping for” and “worried about” to communicate with families. For nurses, we joined staff meetings to describe how to share the graphic aid with families. We also distributed informational brochures and posters with links to online education materials.^32^

### Data Collection

We maintained training logs and competence assessments (eTable 2 in Supplement 1), field notes from on-site observations, meeting minutes, and documentation of conversations with site liaisons and PIs. After implementation, liaisons audited weekly adherence (the number of study-eligible patients and the number of study-eligible patients with an up-to-date BC/WC-ICU graphic aid) and randomly selected up to 3 graphic aids for fidelity evaluation. We used normalization process theory to develop a clinician interview guide, querying respondents about institutional factors, mechanisms or practices that affected implementation, and performance outcomes.^22^ We invited all members of the trauma team for interviews and purposefully selected 48 clinicians from varying roles, capturing 3 to 12 clinicians per site (eTable 3 in Supplement 1). We obtained verbal consent, transcribed audio-recorded interviews, and redacted identifiers. Table 1 displays data collection and the analytic approach used for the RE-AIM outcomes.

**Table 1.** RE-AIM Outcomes With Associated Data Collection and Analytical Approaches

RE-AIM construct	Data collection	Data analysis	Outcome
Reach	Implementation team records (training logs and competence assessments) Weekly adherence audit	Descriptive analysis	Attending/fellow training metrics No. of study-eligible patients % of Eligible patients reached
Effectiveness	Clinician interviews	Deductive content analysis guided by normalization process theory	Effectiveness as reported by clinicians
Adoption	Weekly adherence audit Weekly random sample of graphic aid Clinician interviews Implementation team records (field notes from on-site observation, meeting minutes, conversations with site liaisons and PIs)	Descriptive analysis Formal review of implementation team records and interview transcripts with group consensus	Adherence Graphic aid fidelity Completion on rounds Impact on communication
Implementation	Clinician interviews Implementation team records (field notes from on-site observation, meeting minutes, conversations with site liaisons and PIs)	Deductive and inductive content analysis guided by normalization process theory Formal review of implementation team records and interview transcripts with group consensus	Barriers and facilitators to implementation
Maintenance	Report from sites	Descriptive analysis	Use of intervention beyond 12 mo

Abbreviations: PIs, principal investigators; RE-AIM, Reach, Effectiveness, Adoption, Implementation, and Maintenance.

### Data Analysis

We performed descriptive analysis of training metrics, adherence rates over time, graphic aid fidelity, and maintenance. We estimated the number of patients reached using the number of eligible patients from screening logs and the mean adherence rate during ongoing implementation at each site. We measured fidelity of the graphic aid using an 8-point rubric (eTable 4 in Supplement 1). For missing adherence data, we imputed the mean site adherence when an audit was not completed or when there were no eligible patients. We used Excel version 16.97.2 (Microsoft) for computation and R version 2024.09.0 + 375 (R Foundation) for data presentation.

Four coders (M.L.F., A.H.H., A.B.Z., J.N.) with backgrounds in surgery, education, and sociology used deductive content analysis using codes from normalization process theory and inductive development of new codes not captured by existing constructs.^22^ We mapped codes to the RE-AIM framework to describe the content and relationships around effectiveness and implementation, following the Standards for Reporting Qualitative Research.^33^ To summarize implementation success, we assigned each site a letter grade based on established implementation metrics, including adherence (weekly mean adherence on audit), quality of delivery (graphic aid fidelity, completion on rounds), and completeness of the intervention (clinician delivery of BC/WC-ICU to families).^34,35^

## Results

### Institutional Characteristics

Among study sites, trauma care was delivered in 1 to 3 ICUs, staffed by 8 to 39 trauma ICU attendings and 1 to 25 surgical, anesthesia or pulmonary critical care fellows (eTable 4 in Supplement 1).

### Reach

Initially, we trained 177 trauma surgeons, intensivists, and fellows and an additional 31 individuals through ongoing implementation because of new hires and fellowship turnover. The proportion of attendings and fellows who received individual training at each site ranged from 65% to 100% (eTable 5 in Supplement 1), and 96% achieved competence. The median (IQR) daily number of study-eligible patients ranged from 2 (1-3) to 22 (19-24) at each site. We estimate BC/WC-ICU was delivered to 1354 of 1849 eligible patients (73%).

### Effectiveness

Clinicians reported that BC/WC-ICU helped families comprehend the gravity of their loved one’s injury and see how new events changed expectations related to recovery and prognosis (Table 2). When the best case evolved to include new functional limitations or demonstrated decline, clinicians reported that families would recognize a worsening prognosis. By experiencing this change over time, instead of abruptly confronting news of poor prognosis at a family meeting, the clinical team’s expectations were more transparent, and there was less emotional distress during downstream decisions. One nurse noted, “The family is not like, ‘oh, like, trach, what?’ …it’s been introduced every day at least as a possibility [lessening the] initial shock of those conversations a little bit.” Furthermore, clinicians described that the shared prognostic narrative facilitated consistent communication and produced more harmonious clinician-family relationships.

**Table 2.** Effectiveness of BC/WC-ICU for Patients, Families, and Clinical Teams

Summary	Quotes
Sharing prognostic information using BC/WC-ICU helped families understand severity of illness and long-term trajectory, which prepared them for a prolonged recovery or future decisions.	We’ve had [a] patient [who] has had the same nurse for a couple of days in a row. And [the nurse said]: ‘The family came by yesterday evening, and I showed them the best case worst case. And, you know, they really seem to get it now, how sick grandpa is.’ (Site J – Attending)
	Having those discussions pretty early in his hospital stay helped the family think about it in terms of, you know, what would he actually want? Because, you know, everyone wants their loved one to live, but what does that life look like with the injuries that he has?… Having the tool there to help you facilitate conversations about what the patient enjoyed doing before they were hospitalized really helped frame the family’s thinking about the, you know, whether their dad would want a trach. (Site S – Nurse)
By facilitating reliable and consistent communication, BC/WC-ICU improved relationships between ICU teams and families.	It helps get everybody on the same page… [so] we have a consistent story for the family. Because that’s a huge dissatisfier… when one person presents a trajectory or an overly optimistic or overly pessimistic outcome, and then somebody else provides the opposite of that, that’s very dissatisfying and concerning to families. And I think that, so, you know, level-setting and being on the same page, that helps family members, or that helps patient care. (Site A – Attending)
	I think it helps with communications with the families. I find it, I have been in this job a long time and I’ve found that the communication, like the providers and the families isn’t always stellar… this graphic kind of gives [families] a snapshot of the day. It kind of makes them feel like they’re in the loop, and they know like the main events of the day. And I think that’s been helpful in… kind of improving that, the relationship we have with the patients and families. (Site K – Nurse)
Talking about prognosis as a clinical team supported cohesion within the ICU team and alleviated moral distress.	It gives you like a good update as to like where the patient is in their hospital course, and it kind of like unifies the teams that everybody’s on the same page, like the pharmacists, the dietician, the family members, the residents, the attending, the nurse coordinator. Like we’re all on the same page of like, okay, this person is probably not going to leave the ICU. (Site J – Resident)
	When we do see cases where there isn’t any real improvement in quality of life… we have to just truck on, because it’s not our decision to make. And so, we do get jaded very quickly in the ICU, [we] like know what the end goal would be. (Site J – Nurse) It’s helpful [for]… those longer ICU length of stays [to provide] a frame of reference of this is where you’re going, or this is where you were. And I think that’s been better from a moral injury standpoint and like seeing these patients at all different times. (Site D – Attending)

Abbreviations: BC/WC-ICU, Best Case/Worst Case-ICU; ICU, intensive care unit.

Use of BC/WC-ICU allowed clinicians to routinely reflect on the patient’s illness by departing from solitary clinical abnormalities (eg, anemia, increasing creatinine value) and synthesizing a broader vision around the patient’s health trajectory. Team members felt they could “plan better for the patient” by delineating what it would take to get better or recognizing approaching end of life. When ICU team leaders (attendings or fellows) were not present, nurses, APPs, or residents noted prior completion of BC/WC-ICU allowed them to share critical prognostic information:

We’re translators… when the doctor walks out, [family is] going to ask me what that meant. And so having an actual document that details… if everything goes as good as it possibly can, this is what’s going to happen, and having that be from the attending, is very helpful for the nursing staff. (Site S – Nurse)

Respondents described how BC/WC-ICU mitigated moral distress about patients with poor prognosis. The clinical hierarchy deprives bedside nurses and residents of information about the patient’s longer-term prognosis, leaving doubts about the benefit of ongoing intervention. By increasing access to prognostic information, BC/WC-ICU unified the team’s vision of what was plausible. Clinicians experienced less distress by consistently describing among the team and with family where treatment was headed, even if undesirable (eg, poor neurologic recovery). This alleviated fears that they were prolonging life in a way that was unacceptable to the patient.

### Adoption

**Figure 2.**
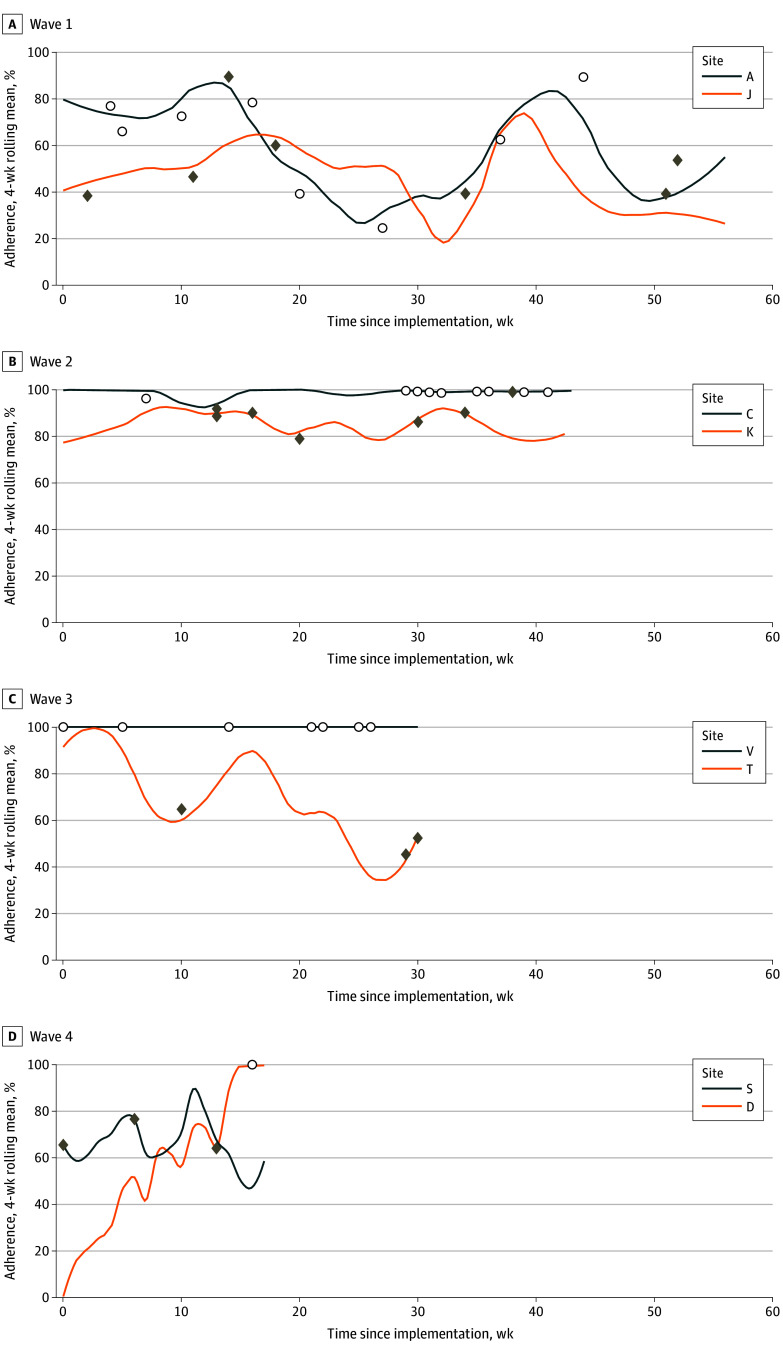
Four-Week Rolling Mean Adherence to Best Case/Worst Case-ICU by Site The adherence, as measured by weekly audit during ongoing implementation for each site, is depicted using a 4-week rolling mean^36^ to demonstrate how adherence fluctuated over time. Sites are stratified by wave to show the variable length of ongoing implementation, which ranged from 56 weeks in wave 1 (earliest implementation training) to 17 weeks in wave 4 (latest implementation training). The open circles indicate weeks during which there were no patients meeting eligibility criteria. The diamonds indicate weeks during which an audit was not completed.

Routine use of BC/WC-ICU varied during ongoing implementation, with mean (SD) weekly adherence ranging from 45% (30.4) to 100% (0) (Figure 2).^36^ Prolonged use was mixed; 3 of 6 sites with longer periods of ongoing implementation demonstrated high routine use for up to 40 weeks. Graphic aid fidelity scores were high, with site mean (SD) scores ranging from 6.22 (2.02) to 7.12 (1.39) out of 8. Early on, we identified concerns when written best-case stories did not address the long-term trajectory of recovery. Thus, we modified the BC/WC-ICU graphic aid to include prompts about important elements (eFigure 3 in Supplement 1). While most sites completed the graphic aid on rounds, this varied, as did clinician delivery of BC/WC-ICU to patient families (Table 3).

**Table 3.** Implementation Outcomes Scorecard Demonstrating Variable Implementation of BC/WC-ICU Across 8 Trauma Centers^a^

Site identifier	Adherence		Quality of delivery score		Completeness of intervention score^c^^,^^d^	Letter grade
	Weekly rate, mean (SD)	% Weeks with 0% adherence	Graphic aid fidelity, mean (SD)^b^	Completion on rounds^c^		
C	98.6 (6.0)	0	6.47 (1.35)	10	9	A+
V	100 (0)	0	7.12 (1.39)	10	5	A
D	60.0 (39.8)	12.5	6.94 (1.46)	7.25	4.5	B+
A	57.3 (44.4)	31.1	6.32 (1.22)	6	6	B
K	84.7 (15.6)	0	6.59 (1.38)	4	4.5	B
J	45.1 (30.4)	18.5	6.43 (1.23)	6	5.75	B-
S	65.8 (25.2)	0	6.57 (1.10)	3	4	C
T	67.6 (25.4)	0	6.22 (2.02)	2	6	C+

Abbreviation: BC/WC-ICU, Best Case/Worst Case-ICU.

^a^ Weekly mean adherence, graphic aid fidelity, completion on rounds, and clinician delivery of BC/WC-ICU to families were equally weighted to calculate a score that was translated into a letter grade. Scores for completion on rounds and clinician delivery of BC/WC-ICU to families were generated from formal review of site-specific field notes wherein the implementation team met as a group to achieve consensus on a score out of 10 points. The percentage of weeks with 0% adherence is included to contextualize the weekly mean adherence but was not included in the final score given significant correlation between high % weeks with 0% adherence and low weekly mean adherence.

^b^ Scale of 0 to 8.

^c^ Scale of 0 to 10.

^d^ Completeness of intervention score refers to clinician delivery of BC/WC-ICU to families.

### Implementation

Implementation of BC/WC-ICU was facilitated by use with all patients (not just study-eligible patients), promoting visibility of the graphic aid to the rounding team, integration of BC/WC-ICU into an ICU checklist, and robust support from liaisons or other on-site champions. Implementation success varied between sites (Table 3). The most successful sites had significant support from dedicated ICU APPs and fewer ICU rounding attendings.

Clinicians reported BC/WC-ICU took 1 to 2 minutes per patient and became easier with practice. Regular trainee turnover required in-the-moment training, making fluidity with the tool difficult. When teams were overwhelmed clinically, time was cited as an insurmountable barrier:

The day we had 21 patients… I had to say, ‘hey, we’re not going to do this today.’ Just because it takes too much time…. Now, the next day, we had a more manageable service, and we did it…. [It] doesn’t take too much time, but it does take a little bit of time. (Site J – Attending)

Rounds and clinical tasks were deemed essential patient care, while BC/WC-ICU was considered extra. Clinicians deprioritized BC/WC-ICU by distinguishing tasks associated with the intervention, including communication with families, from patient care and describing the intervention as an administrative burden: “just another action that we need to get done.”

Clinicians also worried BC/WC-ICU could generate prognostic fear for patients or families. They explained that confronting families with a declining best-case story could be disheartening, and for patients who were doing well, describing the worst-case scenario may cause unnecessary worry. Others shared concerns that documenting the best-case scenario might beget liability as family would view this as a promised outcome rather than a plausible hope. In early implementation, we observed clinicians misunderstanding “best” as expressing a value judgment rather than ordering plausible scenarios. They noted that the best case was for family to transition the patient to comfort care. We swiftly corrected this and in subsequent trainings reinforced that the best-case scenario is the most favorable plausible outcome for the patient’s current treatment, typically life-prolonging care. Finally, the belief that team communication was already sufficient generated a self-reinforcing pathway of ineffective use: token use of the graphic aid had minimal impact on communication with families, reinforcing clinician beliefs that the intervention was not useful.

### Maintenance

After 12 months, 7 sites reported they no longer used the BC/WC-ICU graphic aid. Nonetheless, 4 of the 7 described persistent change in clinician communication, including routine use of “best case” and “worst case” language during family communication. Site C reports ongoing routine use of BC/WC-ICU.

## Discussion

We implemented BC/WC-ICU at 8 trauma centers, where the intervention reached an estimated 1300 seriously injured patients and their families. Clinicians expressed enthusiasm for using BC/WC-ICU to frame prognostic updates with families, allowing them to anticipate additional procedures, functional changes, and longer-term outcomes, thus facilitating downstream decisions. BC/WC-ICU improved team cohesion through consistent messaging, enhanced education of trainees, and diminished moral distress for bedside nurses because prolonged intensive care had a clearly stated plausible goal. Although measures of adherence and fidelity appear remarkable, they do not invariably reflect strong use of the intervention, which was dependent on clinician investment. Simply completing the graphic aid did not ensure the use of scenario planning among ICU teams or in communication with families.

Routine use of BC/WC-ICU required persistent engagement by the implementation team and site champions. Consistent with the framework of normalization process theory, which recognizes interventions as a combination of beliefs and practices,^22^ we observed effective implementation was self-reinforcing; when clinicians regularly completed BC/WC-ICU they found it useful. Yet without early adoption and visible demonstration of the intervention’s utility, team engagement lagged, and use of BC/WC-ICU faded. Despite general enthusiasm for this intervention from clinicians, institutions, and across surgery more broadly,^15,16,17,18^ our findings suggest that the model of communicating through scenario planning and a graphic aid may not be sustainable without stronger incentives. Our study has important implications for families and patients, clinicians, and health systems.

For families and patients, BC/WC-ICU can be used to convey prognostic information, which clinicians avoid despite the importance of this information for downstream decisions.^37,38^ It is unclear which aspects of the intervention are most impactful. Articulating expectations about duration of treatment, functional outcomes, and length of survival can support imagination about new health states, adaptation to clinical reality, and reckoning with mortality in a way that reporting a disarticulated list of clinical problems or isolated statistics cannot.^39,40,41^ Using the language of “we are hoping for…” and “we are worried about…” can demonstrate uncertainty within the context of plausibility and hope.^42,43^ Establishing unity around prognosis during rounds can empower clinicians to convey a consistent message about prognosis to families, even when families are not present during BC/WC-ICU completion. Despite these benefits, our respondents noted prognostic awareness can be upsetting, and others report that patients prefer clinician optimism.^44^ Clinicians are in a difficult bind as they seek to provide information to facilitate goal-concordant care when this information is painful to receive.^45^ To this end, BC/WC-ICU is not a stand-alone intervention. While information is often prioritized over addressing the emotional valence of serious injury care, this intervention needs to be delivered carefully, with attention to emotion and sensitivity about when to stop delivering information people are not ready to hear.^46,47,48,49^

For clinicians, changing communication is difficult. Despite prolonged educational efforts, obvious prompts on rounds, and regular advocacy from local champions, BC/WC-ICU failed when it did not become entrenched. Existing patterns of sporadic clinician-family communication were difficult to alter. Specifically, bedside nurses were the singular source of updates unless there was a transformative patient event, which reduced access to important prognostic information. While it is natural for kind and caring clinicians to believe they are good communicators,^50^ advanced communication skills are often overlooked and regularly underused.^51,52^ Paradoxically, diligent and thoughtful clinicians pose a major implementation barrier; despite describing preexisting local challenges with communication, limited insight into gaps in personal performance makes practice change difficult.

For health systems, improving clinician communication will require fundamental change. As clinicians are asked to “do more with less” with high patient acuity and resource constraints,^53,54^ they deprioritize communication and BC/WC-ICU by considering this an administrative task. While some clinicians viewed communication with families as adjunct rather than integral to patient care, many described aspirational commitments to patient and family engagement that buckled under the weight of daily clinical work; communication was reduced to the smallest amount necessary to provide evidence-based care.^55^ Despite the best intentions of individuals, systems that reward intervention, technological advancement, and high throughput without incentivizing routine use of advanced communication—not just for the sickest patients or in moments of great transition—are unlikely to improve.

### Strengths and Limitations

Our study has strengths and limitations. Weekly adherence reports use rates for cross-site comparison, yet this measure is skewed by the number of study-eligible patients, eg, 60% adherence in a busy ICU represents greater use than 100% with only 1 study-eligible patient. Access to the direct experience of families was unavailable for this study in part due to the difficulty identifying who received BC/WC-ICU. Clinician reporting provides some insight into how BC/WC-ICU impacted clinician-family dynamics, yet our knowledge of the patient and family viewpoint is confined to enthusiasm from patient and family trauma survivors in the Coalition for National Trauma Research Injury Research Engagement Project, whose advisory support and feedback throughout this study aligned with clinician observations.

## Conclusions

We successfully implemented BC/WC-ICU at 8 major trauma centers, which clinicians perceived supported families in their understanding of the difficult experience of serious injury care. It also supported clinical team cohesion and reduced moral distress by delivering consistent prognostic information for more than 1300 patients. Despite these successes, implementation was challenged by the fast pace and difficult work of providing intensive care for many seriously injured patients and ambivalence about the relationship between communication and patient outcomes. Successful implementation of advanced communication strategies will require rethinking the motivations and support for clinical teams to change practice.

## Notes

Supplement 1. **eTable 1.** Guide to using Best Case/Worst Case-ICU as an ICU team with suggestions for participation according to clinical role **eTable 2.** Rubric for competence evaluation of attendings and fellows after completing one-on-one Best Case/Worst Case-ICU training **eTable 3.** Demographics and characteristics of clinician interviewees **eTable 4.** Fidelity checklist for evaluation of the Best Case/Worst Case-ICU (BC/WC-ICU) graphic aids **eTable 5.** Variation in institutional characteristics and implementation context across eight trauma centers **eFigure 1.** Feedback messaging delivered to trauma centers using positive feedback strategy to encourage ongoing use **eFigure 2.** Feedback messaging delivered to trauma centers using coaching to reinforce effective completion of the graphic aid and suggest opportunities for improvement **eFigure 3.** Modified Best Case/Worst Case-ICU graphic aid with template to prompt best case story elements

Supplement 2. Data sharing statement
