# Supplementary material for: Best Case/Worst Case Communication Tool for Trauma Intensive Care Units
Source: JAMA Surg. 2025 Sep 24;160(11):1250–9. doi: 10.1001/jamasurg.2025.3782 (PMC12461598; doi:10.1001/jamasurg.2025.3782)
Supplement: Supplement 1. — eTable 1. Guide to using Best Case/Worst Case-ICU as an ICU team with suggestions for participation according to clinical role eTable 2. Rubric for competence evaluation of attendings and fellows after completing one-on-one Best Case/Worst Case-ICU training eTable 3. Demographics and characteristics of clinician interviewees eTable 4. Fidelity checklist for evaluation of the Best Case/Worst Case-ICU (BC/WC-ICU) graphic aids eTable 5. Variation in institutional characteristics and implementation context across eight trauma centers eFigure 1. Feedback messaging delivered to trauma centers using positive feedback strategy to encourage ongoing use eFigure 2. Feedback messaging delivered to trauma centers using coaching to reinforce effective completion of the graphic aid and suggest opportunities for improvement eFigure 3. Modified Best Case/Worst Case-ICU graphic aid with template to prompt best case story elements [file jamasurg-e253782-s001.pdf]

Supplemental Online Content

Fritz ML, Hernandez AH, Zelenski AB, et al. Best case/worst case communication tool for trauma intensive care units. *JAMA Surg*. Published online September 24, 2025. doi:10.1001/jamasurg.2025.3782

eTable 1. Guide to using Best Case/Worst Case-ICU as an ICU team with suggestions for participation according to clinical role

eTable 2. Rubric for competence evaluation of attendings and fellows after completing one-on-one Best Case/Worst Case-ICU training

eTable 3. Demographics and characteristics of clinician interviewees

eTable 4. Fidelity checklist for evaluation of the Best Case/Worst Case-ICU (BC/WC-ICU) graphic aids

eTable 5. Variation in institutional characteristics and implementation context across eight trauma centers

eFigure 1. Feedback messaging delivered to trauma centers using positive feedback strategy to encourage ongoing use

eFigure 2. Feedback messaging delivered to trauma centers using coaching to reinforce effective completion of the graphic aid and suggest opportunities for improvement

eFigure 3. Modified Best Case/Worst Case-ICU graphic aid with template to prompt best case story elements

This supplemental material has been provided by the authors to give readers additional information about their work.

© 2025 Fritz ML et al. *JAMA Surgery*.

eTable 1. Guide to using Best Case/Worst Case-ICU as an ICU team with suggestions for participation according to clinical role.

Task	Recommended Clinical Role	Guidance for Completion
Generating the outlook	Attending or Fellow	*It is important that a team member with experience generates the outlook: they need to use their experience and knowledge to tell the story of the Best Case and Worst Case Scenarios. The attending or fellow may want to invite a resident to generate the outlook and then confirm or adjust the story. If the team member leading the systems-based patient presentation has the experience to generate the outlook, they can do so by starting, "In the Best Case Scenario, we are hoping…" If the team member leading the systems-based patient presentation does not feel that they have the experience to generate the outlook, they can prompt the attending/fellow to describe the outlook at the end of the review by saying "What is the outlook?"*
Annotating the graphic aid	Resident or APP	*Someone on the team will be responsible for writing the patient's name, room number, and date on the graphic aid. They will also list any overnight events, draw the star related to prognosis, and write bullet points next to the star to briefly convey the Best Case Scenario to family members. If they have questions about what overnight events should be listed, or how to represent the Best Case Scenario with bullet points on the graphic aid, they should ask the person who is generating the outlook.*
Confirming graphic aid notes with the team	Resident or APP	*It may be helpful to pause after generating the outlook and review the graphic aid as a team to ensure everyone is on the same page. The team can confirm the location of the star (Best Case Scenario) in relation to the square (Worst Case Scenario), what event(s) are listed at the top of the day, and what bullet points describing the Best Case Scenario are listed next to the star.*
Using the graphic aid with patients and families/loved ones	Any team member	*If family is at the patient's bedside, the physicians or APPs caring for the patient should use the graphic aid during their daily communication with family members. If daily updates with family occur over the phone, it is still useful to describe the Best and Worst Case Scenarios. Remember that the Best Case Scenario describes "what we are hoping for" and the Worst Case Scenario describes "what we are worried about". Discussing the graphic aid with families is not limited to physicians or APPs. Nurses, therapists, pharmacists, and other team members can all use the graphic aid to discuss the patient's care or outlook with their family members. If the family has additional questions about the graphic aid any team member can reach out to resident physicians, APPs, or attending physicians to further discuss the graphic aid with the family.*
Filling out the back side of the graphic aid	Patients, family members, any team member	*Family members can fill out the back side of the graphic aid to share more about their loved one. Anyone on the team or who participates in the patient's care can encourage the family to complete this and assist them if needed.*

© 2025 Fritz ML et al. *JAMA Surgery*.

eTable 2. Rubric for competence evaluation of attendings and fellows after completing one-on-one Best Case/Worst Case-ICU training.

**Items**	**Score**
**Best Case Scenario (5)**	**0/1**[a]
Told a story about what we are hoping for with a **beginning, middle and end**	
Story covered the anticipated amount of **time** in ICU, hospital, post-acute care	
Included information about additional **treatments** – eg ventilator, surgery, physical therapy	
Included details about where the patient would **live** after recovery (6-12 months from now)	
Described **functional status** "after recovery"	
**Worst Case Scenario (1)**	
Clearly stated the worst case scenario – including the possibility of death if appropriate	
**Identify Major Events (2)**	
Named an event from the previous day or stated that there were no new events	
Explained how event(s) changed the Best Case Story	
**Fill out the GA (3)**	
Wrote an **event** (or Ø) on the GA	
Placed the Best Case **Star** on the GA	
Wrote **bullet points/notes** on the GA	
**Talk with patients and family members using the GA (3)**	
Referred to the GA when talking to "family member"	
Used phrases like "We are **hoping for**…"	
Used phrases like "We are **worried about**…"	
**Total Score**[b]	

a. Participants received 1 point for each component of the tool that they successfully completed. If they did not successfully complete that component, they received 0 points. If you have to prompt the learner, score 0 and make a note that you prompted them.
b. Final scores were out of 14 possible points. A score of ≥10 was prespecified as indicating competence

© 2025 Fritz ML et al. *JAMA Surgery*.

eTable 3. Demographics and characteristics of clinician interviewees.

Characteristic	All interviewees, n=46[a]
Age, mean (SD), years	39.9 (10.3)
Years in current line of work, mean (SD)	9 (8.3)
Clinician Role, n (%)	
Attending Physician	15 (32.6)
Advanced Practice Provider (Nurse Practitioner or Physician Assistant)	8 (17.4)
Fellow	4 (8.7)
Nurse	11 (23.9)
Resident	4 (8.7)
Other (Provider, Assistant Nurse Manager, Registered Dietitian, No Response)	4 (8.7)
Gender, n (%)	
Female	29 (63.0)
Male	17 (37.0)
Race[b], n (%)	
White	38 (84.4)
Asian	44 (8.7)
Black or African American	3 (6.5)
Unknown/Not Reported	1 (2.2)
None selected	2 (4.3)
Ethnicity, n (%)	
Hispanic or Latino	4 (8.7)
Not Hispanic or Latino	41 (89.1)
Omitted	1 (2.2)
Hospital Site, n (%)	
A	8 (17.4)
C	5 (10.9)
D	3 (6.5)
J	11 (23.9)
K	8 (17.4)
S	3 (6.5)
T	3 (6.5)
V	5 (10.9)

a. We completed interviews with 48 clinicians, of whom 46 completed demographic surveys.
b. Percentages do not total 100% as some individuals selected multiple racial identifiers.

© 2025 Fritz ML et al. *JAMA Surgery*.

eTable 4. Fidelity checklist for evaluation of the Best Case/Worst Case-ICU (BC/WC-ICU) graphic aids.[a]

	0 points	1 point	2 points
**Graphic Aid Elements**			
**Star**	No stars are marked on GA	Star is present for some, but not all, of the evident days.[b]	Star is present for all of the evident days.
**Event**	No events[c] are marked on GA	Event is present for some, but not all, of the evident days.	Event is present for all of the evident days.
**Quality of Notations**			
**Best Case Scenario**	No written best case	Written notes with 2 or less of the following:<br>- Treatments needed<br>- Units of time<br>- Where the patient will live after leaving the hospital<br>- Functional status in the future	Written notes with 3 or more of the following:<br>- Treatments needed<br>- Units of time<br>- Where the patient will live after leaving the hospital<br>- Functional status in the future
**Worst Case Scenario**	No written worst case	Worst case includes one element (e.g. death)	Worst case includes two or more elements (e.g. needs more procedures, gets sicker, needs more help going forward)

a. Site liaisons randomly selected three graphic aids on a weekly basis and uploaded these for evaluation of fidelity by the UW-based implementation team.
b. “Evident days” were columns in which there was some written marking indicating that the team intended to or started to complete BC/WC-ICU on that date.
c. “No event” or “n/a” or a circle with line through it written on the graphic aid was taken as an indication that there was no major event. These notations were considered “events” for the purpose of scoring.

© 2025 Fritz ML et al. *JAMA Surgery*.

eTable 5. Variation in institutional characteristics and implementation context across eight trauma centers.

Site ID	# Eligible Patients on Audit Median (IQR)	# ICUs	# Attendings	# Fellows	% Attendings/Fellows Trained	# of Specialties Represented by Attendings
A	2 (1-3)	2	18	3	100%	1
C	4 (2-5)	1	10	3	100%	1
D	3 (2-5)	2	19	5	96%	1
J	5 (4-6)	2	39	16	65%	4
K	10 (8-13)	3	20	25	78%	5
S	10 (8-11)	3	15	4	100%	1
T	22 (19-24)	3	14	8	86%	1
V	2 (1-3)	1	8	1	100%	1

© 2025 Fritz ML et al. *JAMA Surgery*.

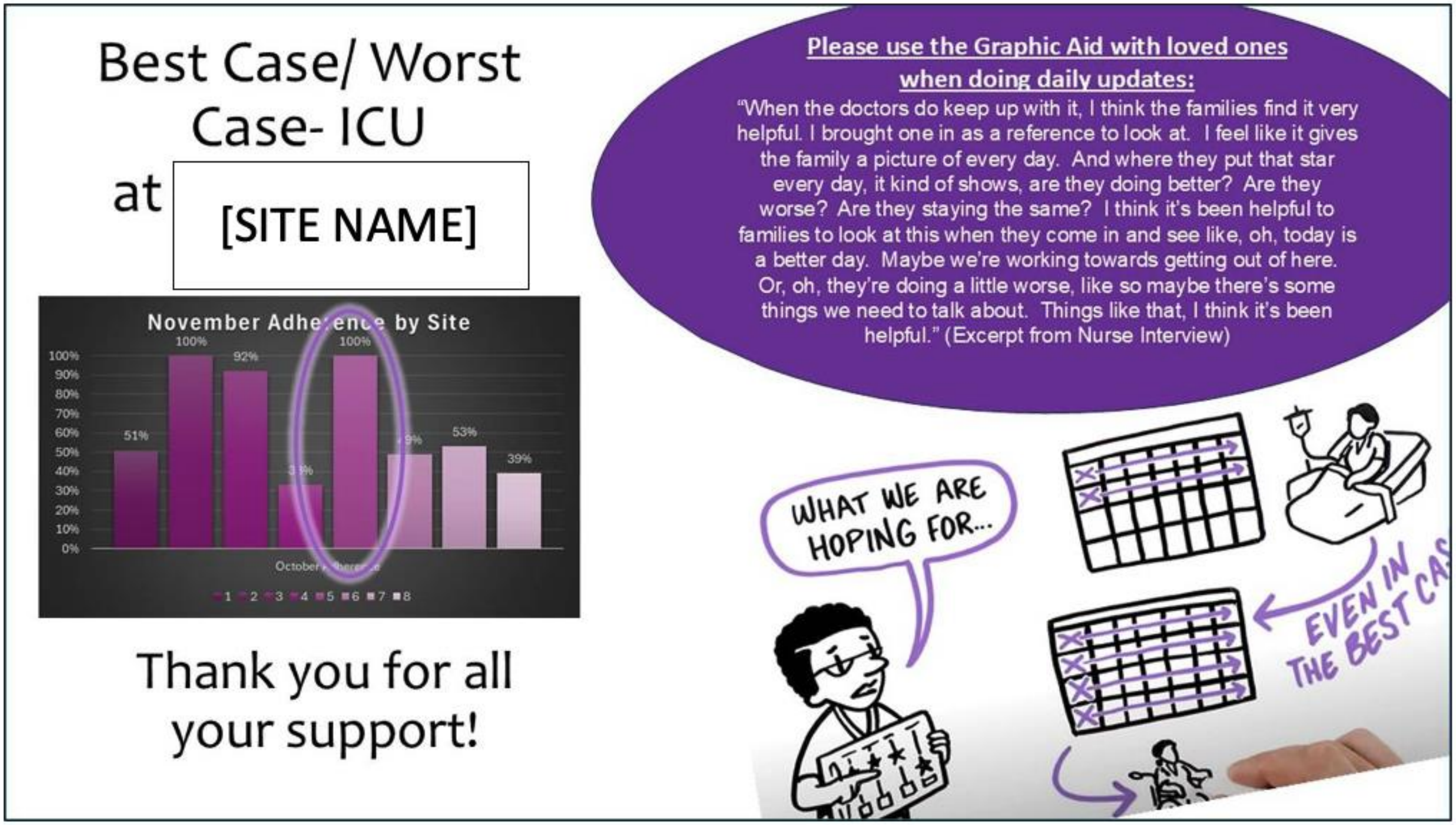

eFigure 1. Feedback messaging delivered to trauma centers using positive feedback strategy to encourage ongoing use.

© 2025 Fritz ML et al. *JAMA Surgery*.

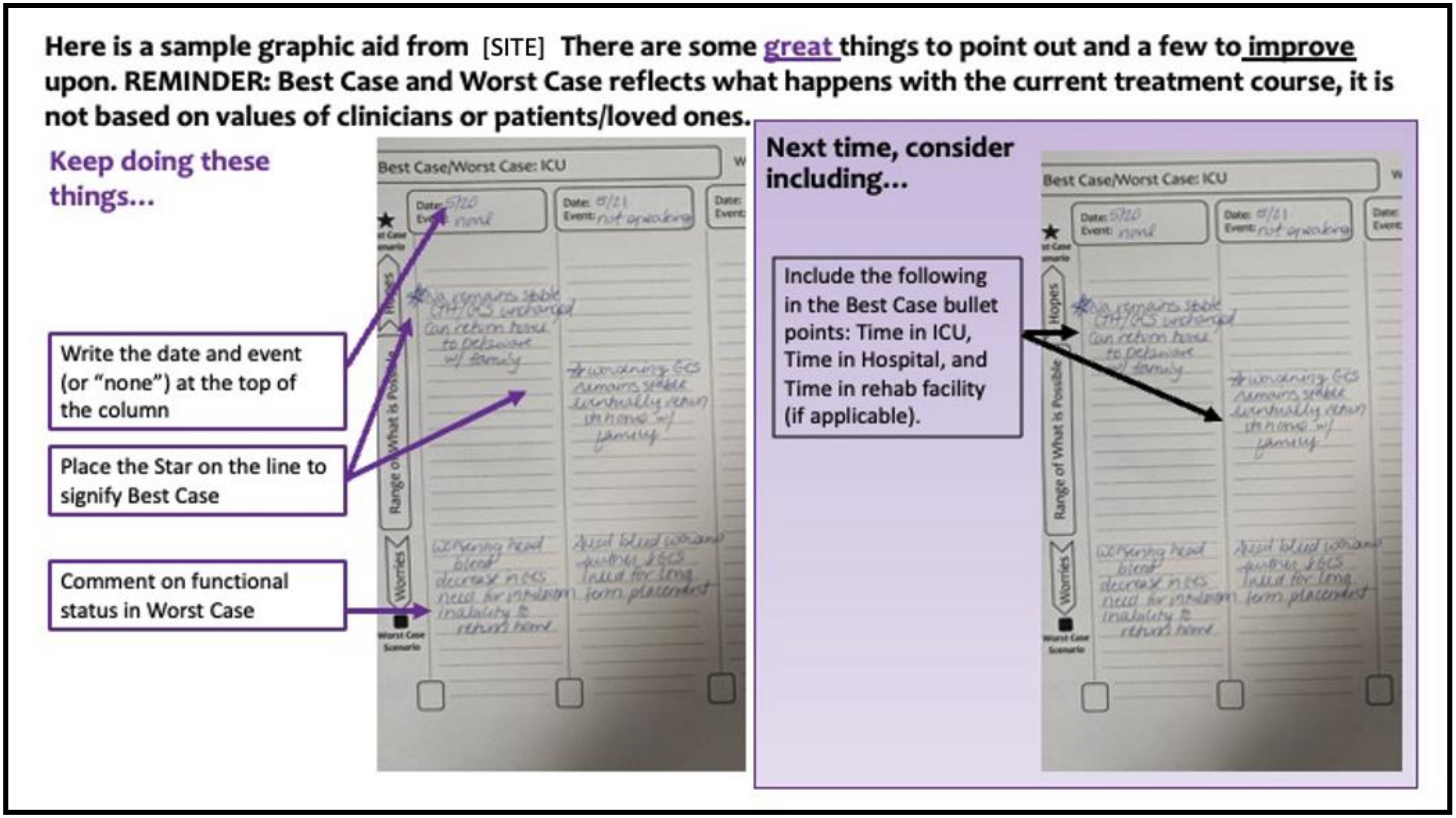

eFigure 2. Feedback messaging delivered to trauma centers using coaching to reinforce effective completion of the graphic aid and suggest opportunities for improvement.

© 2025 Fritz ML et al. *JAMA Surgery*.

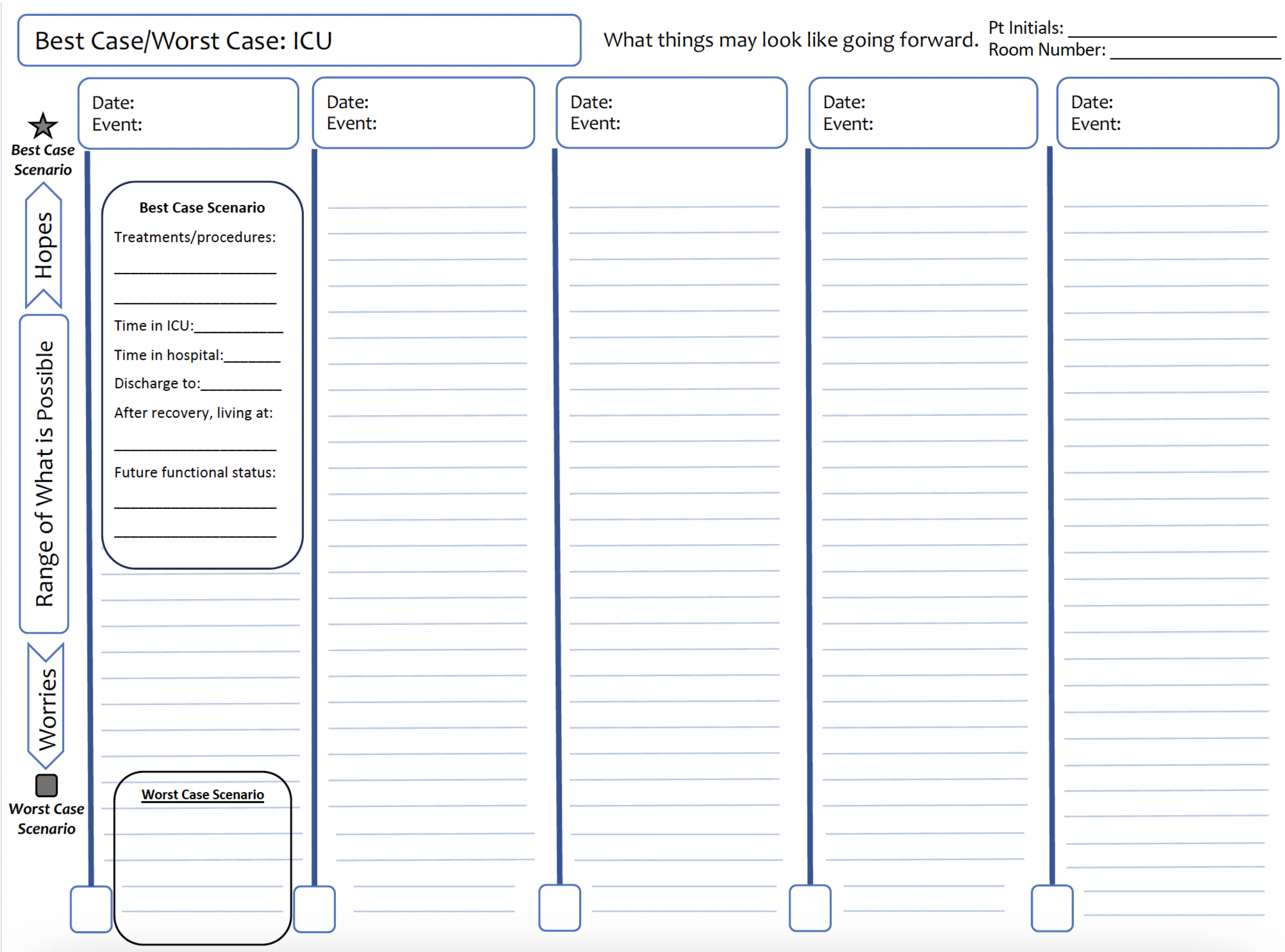

eFigure 3. Modified Best Case/Worst Case-ICU graphic aid with template to prompt best case story elements.

© 2025 Fritz ML et al. *JAMA Surgery*.
