# Supplementary material for: Best Case/Worst Case Communication Tool for Trauma Intensive Care Units
Source: JAMA Surg. 2025 Sep 24;160(11):1250–9. doi: 10.1001/jamasurg.2025.3782 (PMC12461598; doi:10.1001/jamasurg.2025.3782)
Supplement: Supplement 2. — Data sharing statement [file jamasurg-e253782-s002.pdf]

Data Sharing Statement

Fritz. Best Case/Worst Case Communication Tool for Trauma Intensive Care Units. *JAMA Surg*. Published September 24, 2025. doi:10.1001/jamasurg.2025.3782

Data
Data available: No

Additional Information
Explanation for why data not available: Interview data is not available for future research purposes. The study team will consider select requests to share de-identified adherence and fidelity data.
